# Multiple myeloma induces Mcl-1 expression and survival of myeloid-derived suppressor cells

**DOI:** 10.18632/oncotarget.3300

**Published:** 2015-03-23

**Authors:** Kim De Veirman, Jo A. Van Ginderachter, Susanne Lub, Nathan De Beule, Kris Thielemans, Ivan Bautmans, Babatunde O. Oyajobi, Elke De Bruyne, Eline Menu, Miguel Lemaire, Ivan Van Riet, Karin Vanderkerken, Els Van Valckenborgh

**Affiliations:** ^1^ Laboratory of Hematology and Immunology, Myeloma Center Brussels, Vrije Universiteit Brussel (VUB), Brussels, Belgium; ^2^ Laboratory of Cellular and Molecular Immunology, Vrije Universiteit Brussel, Brussels, Belgium; ^3^ Myeloid Cell Immunology Laboratory, VIB, Brussels, Belgium; ^4^ Department of Immunology-Physiology, Laboratory of Molecular and Cellular Therapy, Vrije Universiteit Brussel, Brussels, Belgium; ^5^ Gerontology & Frailty in Ageing Departments, Vrije Universiteit Brussel, Brussels, Belgium; ^6^ Department of Cellular & Structural Biology and Cancer Therapy and Research Center, The University of Texas Health Science Center at San Antonio, San Antonio, USA

**Keywords:** multiple myeloma, myeloid-derived suppressor cells, generation, mechanism, targeting

## Abstract

Myeloid-derived suppressor cells (MDSC) are contributing to an immunosuppressive environment by their ability to inhibit T cell activity and thereby promoting cancer progression. An important feature of the incurable plasma cell malignancy Multiple Myeloma (MM) is immune dysfunction. MDSC were previously identified to be present and active in MM patients, however little is known about the MDSC-inducing and -activating capacity of MM cells. In this study we investigated the effects of the tumor microenvironment on MDSC survival. During MM progression in the 5TMM mouse model, accumulation of MDSC in the bone marrow was observed in early stages of disease development, while circulating myeloid cells were increased at later stages of disease. Interestingly, *in vivo* MDSC targeting by anti-GR1 antibodies and 5-Fluorouracil resulted in a significant reduced tumor load in 5TMM-diseased mice. *In vitro* generation of MDSC was demonstrated by increased T cell immunosuppressive capacity and MDSC survival was observed in the presence of MM-conditioned medium. Finally, increased Mcl-1 expression was identified as underlying mechanism for MDSC survival. In conclusion, our data demonstrate that soluble factors from MM cells are able to generate MDSC through Mcl-1 upregulation and this cell population can be considered as a possible target in MM disease.

## INTRODUCTION

Multiple Myeloma (MM) is an incurable B-cell malignancy characterized by clonal proliferation of malignant plasma cells within the bone marrow (BM). Clinical features of this disease are anemia, renal failure, hypercalcemia and osteolytic bone lesions. In addition, immunodeficiency is a typical observation in patients with active MM [1]. MM cells evade immunosurveillance through several mechanisms including the generation of immunosuppressive cell types (regulatory T cells, Th17 cells and myeloid-derived suppressor cells), secretion of immune-suppressive molecules, and abnormalities in number and function of macrophages, T cells, B cells, natural killer T cells and dendritic cells [2–4]. The immune deficiency not only contributes to the susceptibility to infections, but also plays a crucial role in disease pathogenesis and progression [4]. In the past decade, immunomodulatory drugs (IMiDs) including thalidomide, lenalidomide and recently pomalidomide, emerged as a new treatment option in MM patients. Besides a direct anti-MM effect, these agents modulate the BM microenvironment and enhance the host immune response. Importantly, the combination of IMiDs with other anti-MM drugs dramatically increases the outcome of patients with MM [5]. However, MM is still incurable and most of the patients relapse. Thus, unraveling of relapse and resistance mechanisms is necessary.

It has become clear that myeloid-derived suppressor cells (MDSC) play a major role in immunosuppression in cancer [6–9]. MDSC form a heterogeneous population of immature myeloid cells with strong T cell suppressive activities. They accumulate during cancer, inflammation and infection in the blood, lymph nodes, BM and at tumor sites in human cancers and animal models. In mice, MDSC are characterized by the dual expression of CD11b and Gr-1. In addition, these cells can be subdivided into monocytic (CD11b^+^Ly6G^−^Ly6C^high^) and granulocytic (CD11b^+^Ly6G^+^Ly6C^low^) populations [9, 10]. Among human patients, MDSC are highly heterogeneous in antigen expression and are generally defined based on the expression of CD11b and CD33, and a lack of the mature marker HLA-DR. Furthermore, monocytic MDSC tend to be more CD14^+^, while granulocytic myeloid cells are CD15^+^ [10–12]. However, there is no clear consensus about the phenotypic characterization of both populations in humans.

Extensive data exist on the MDSC suppressive mechanisms in murine models. MDSC suppress the immune system by production of arginase-1, nitric oxide synthase, reactive oxygen species (ROS), immunosuppressive cytokines (IL-6, IL-10) and regulatory T cell activation [4]. However, few data are available about the underlying mechanisms of generation and activation of MDSC in the context of cancer. A range of soluble factors such as GM-CSF, G-CSF, M-CSF, IL-6, IL-10, VEGF and IL-1β are described to stimulate MDSC accumulation [10, 13, 14]. Also, STAT3 has been identified as the main transcription factor for MDSC expansion while STAT1 and STAT6 are stimulators of MDSC function [15, 16]. Increased STAT3 phosphorylation was observed in MDSC of tumor-bearing mice compared to immature myeloid cells of naive mice [17]. Furthermore, STAT3 activation is associated with increased survival of myeloid cells by inducing the expression of STAT3 target genes including Bcl-xL, Myc, survivin, cyclin D1 and calcium-binding pro-inflammatory proteins S100A8 and S100A9 [10].

Recent studies described the presence and immunosuppressive capacity of monocytic and granulocytic MDSC in MM mouse models [18–20]. Also in MM patients both MDSC populations have been described. A significant increase was observed of CD11b^+^CD33^+^CD14^−^CD15^+^ (granulocytic) MDSC in the blood and BM of MM patients [18, 19, 21]. Although no change in the monocytic (CD14^+^HLA-DR^low^) cell population was observed, both subgroups exert immunosuppressive activities [19]. One study describes the importance of S100A9 in the accumulation of MDSC in MM and blocking MDSC accumulation could delay the development of MM tumor [19]. No other underlying mechanisms of MDSC accumulation are described in MM.

The aim of our study was to investigate the development of MDSC during myeloma progression and its importance in MM growth using the 5TMM mouse models. We assessed the role of MM soluble factors on MDSC survival and accumulation and further defined the underlying mechanisms, focusing on STAT3 and bcl-2 family proteins.

## RESULTS

### MDSC distribution in bone marrow, blood and spleen of 5T33MM mice during disease progression

**Figure 1:**
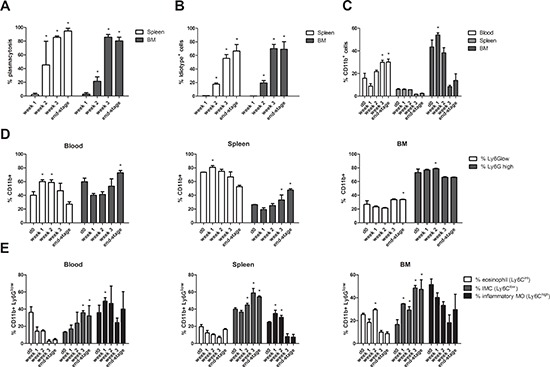
MDSC distribution in the bone marrow, blood and spleen of 5T33MM mice during disease progression C57BL/KaLwRij mice were inoculated with 5T33MMvv cells and sacrificed at one, two and three weeks after inoculation and at the end-stage of the disease (*n* = 3/group). Blood, BM and spleen were investigated. **A.** % Plasmacytosis determined by microscopic examination of cytospins stained by the May-Grünwald-Giemsa method. **B.** % Idiotype+ cells detected by anti-idiotype (3H2) FACS staining to detect tumor load. **C.** The percentage of CD11b^+^ cells (gated on 3H2^−^ cells) was determined by flow cytometry. **D.** Ly6G expression in the CD11b^+^ population was analyzed by flow cytometry. **E.** In the CD11b^+^ Ly6G^low^ population, Ly6C expression was analyzed by flow cytometry to distinguish inflammatory monocytes (MO) (Ly6C^hi^), eosinophils (Ly6C^int^), and immature myeloid cells (IMC) (Ly6C^low^). Error bars represent the SD. * indicates *p* < 0.05 and represents the significant increase compared to week 1 (Figure 1A and 1B) or day 0 (Figure 1C and 1E).

C57BL/KaLwRij mice were inoculated with 5T33MMvv cells and sacrificed one, two and three weeks after inoculation and at end-stage of the disease. Tumor load was assessed by microscopic examination of cytospins (Figure 1A) and anti-idiotype FACS staining (Figure 1B) of isolated BM. The presence of CD11b^+^ cells, gated on the idiotype negative cell population (3H2^−^), was investigated by flow cytometry and we observed a significant increase in the BM one week after MM cell inoculation. At later stages of disease a decrease in the BM could be observed, while circulating myeloid cells increased (Figure 1C). In the spleen, we observed a low percentage of CD11b^+^ cells with no significant changes during MM progression.

We examined the presence of Ly6G^low^ (monocytic) versus Ly6G^high^ (granulocytic) cells within the CD11b^+^ population of blood, spleen and bone marrow at different stages of MM progression (Figure 1D). During disease progression, an early increase of Ly6G^low^ cells in the blood and spleen that switches to an increased Ly6G^high^ population at the end-stage of the disease was observed, while no clear switches in the abundance of bone marrow MDSC populations could be seen. However, within the CD11b^+^Ly6G^low^ cell population, three distinct subtypes can be discriminated based on Ly6C expression: (a) Ly6C^hi^ inflammatory monocytes (MO), (b) Ly6C^int^ eosinophils, and (c) Ly6C^low^ immature myeloid cells (IMC) (gating strategy shown in Supplementary Figure 1), all of which were reported to possess immunosuppressive activity [20]. Interestingly, an increase in the IMC population in blood, spleen and bone marrow could be observed during disease progression, suggesting an overall myeloid cell differentiation block in the presence of MM cells (Figure 1E).

### MDSC depletion by anti-GR1 antibodies and 5-Fluorouracil *in vivo*

**Figure 2:**
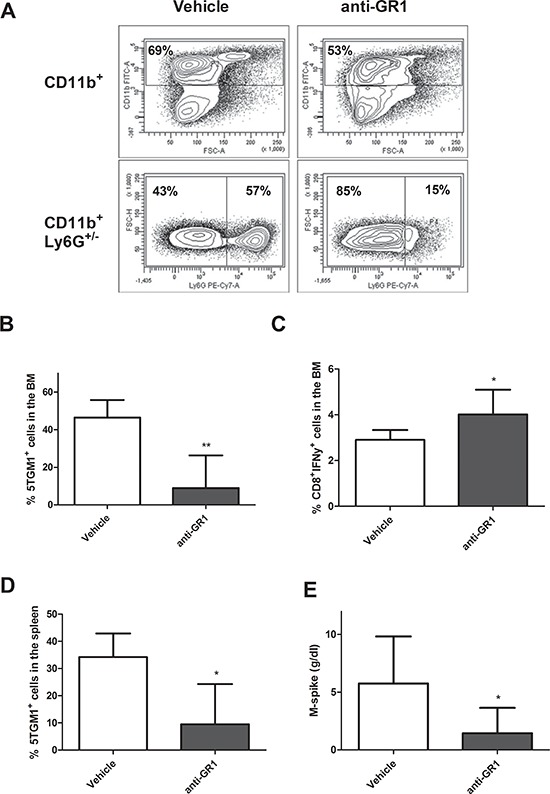
*In vivo* MDSC targeting by anti-GR1 **A.** Naive mice were treated with 200 μg/mL anti-GR1 antibody and sacrificed two days later. The percentage CD11b^+^ and Ly6G^+^ cells were analyzed by flow cytometry (*n* = 2). **B–E.** Mice were inoculated with 5TGM1-GFP^+^ cells and treated with vehicle (*n* = 5) or anti-GR1 antibodies (*n* = 7) (200 μg/mL, every two days) for 4 weeks. The effect on tumor load in the BM and spleen and IFNγ secreting CD8^+^ T cells in the BM was assessed by flow cytometry. M-spike was measured by means of serum electrophoresis. * indicate *p* < 0.05, ** indicate *p* < 0.01 (Mann–Whitney *U*-test). Error bars represent the SD.

We next investigated whether MDSC depletion can influence tumor development by depleting these cells using an anti-GR1 antibody. As 5T33MMvv-derived 5TGM1 cells were negative for GR1 expression, we preferred this model for *in vivo* MDSC targeting. Since we observed already an early accumulation of CD11b+ cells in MM mice, we initiated treatment with anti-GR1 antibodies one day after inoculation. Therefore, we first checked the effect of anti-GR1 antibodies on the CD11b^+^ population in naive mice. Two days after antibody administration, we observed a reduction in total CD11b^+^ cell number, mainly by depletion of the Ly6G^+^ (granulocytic) population in the BM (Figure 2A). Hence, one day after injection of 5TGM1 cells, mice were treated with anti-GR1 antibodies during 5 weeks and tumor load was assessed when mice showed signs of disease. A significant reduction in 5TGM1-GFP^+^ cells in the BM, accompanied by an upregulation in IFNγ-secreting CD8^+^ T cells was observed (Figure 2B–2C), along with a diminished tumor load in the spleen and reduced serum M-spike (Figure 2D–2E).

**Figure 3:**
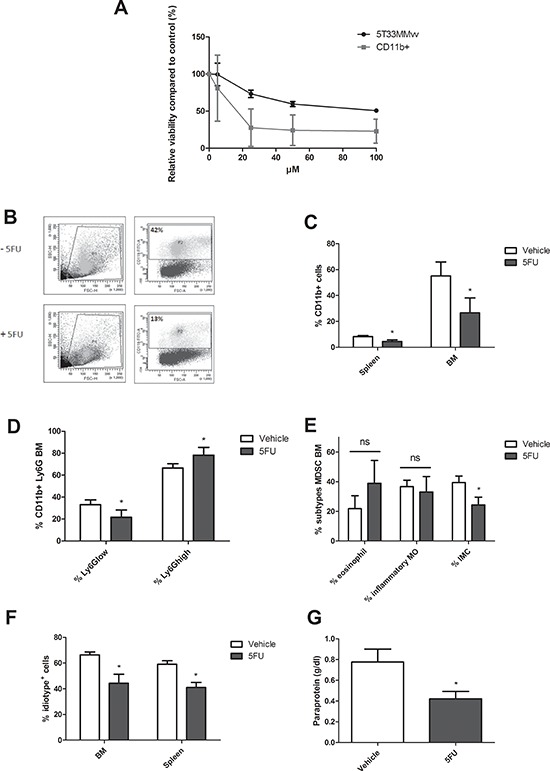
*In vivo* MDSC targeting by 5-Fluorouracil **A.** 5T33MMvv cells and CD11b^+^ cells were treated with increasing concentrations of 5FU for 48 h and analyzed for viability by CellTiter-Glo assay (*n* = 3). **B–C.** 5T33MM mice were treated with 50 mg/kg of 5-fluorouracil (5FU). Mice were sacrificed four days after drug treatment. The percentage CD11b+ cells in the BM and spleen was determined by flow cytometry (*n* = 4). **D.** Ly6G expression in the BM analyzed by flow cytometry (*n* = 4). **E.** In the CD11b^+^Ly6G^low^ population three MDSC subtypes were distinguished based on Ly6C (Ly6C^hi^ inflammatory monocytes (MO), Ly6C^int^ eosinophils, and Ly6C^low^ immature myeloid cells (IMC)) (*n* = 4). **F–G.** 5T33MM mice were treated with 50 mg/kg of 5-fluorouracil (5FU) (one dose, i.p., 4 days after 5T33MMvv cell inoculation) and sacrificed 17 days after MM cell inoculation (*n* = 3/group). Tumor load was assessed by M-spike detection and 3H2 (anti-idiotype) FACS staining. * indicate *p* < 0.05 (Mann–Whitney *U*-test). Error bars represent the SD.

In addition, we evaluated the effect of the chemotherapeutic agent 5-fluorouracil (5FU), a pyrimidine analog with MDSC depleting capacity. *Ex vivo*, 5T33MMvv cells (CD11b^−^) and MDSC (CD11b^+^) were incubated with increasing concentrations of 5FU. We observed a reduction in cell viability of both populations, however the CD11b^+^ cells were much more sensitive to 5FU (IC50 5T33MMvv = 84.5 μM, IC50 CD11b^+^ = 15.1 μM) (Figure 3A). *In vivo*, 5FU administration resulted in a reduction of CD11b^+^ cells in the BM and spleen (Figure 3B and 3C). Further analysis demonstrated that the Ly6G^low^ subpopulation is affected by 5FU, with mainly an effect on immature myeloid cells (Figure 3D and 3E). Importantly, 5FU administration reduced M-spike and idiotype positive cells in the BM and spleen (Figure 3F and 3G).

All these data indicate that both monocytic and granulocytic MDSC are active in MM disease and that targeting MDSC inhibits tumor development.

### Generation of murine MDSC in myeloma cell conditioned medium

**Figure 4:**
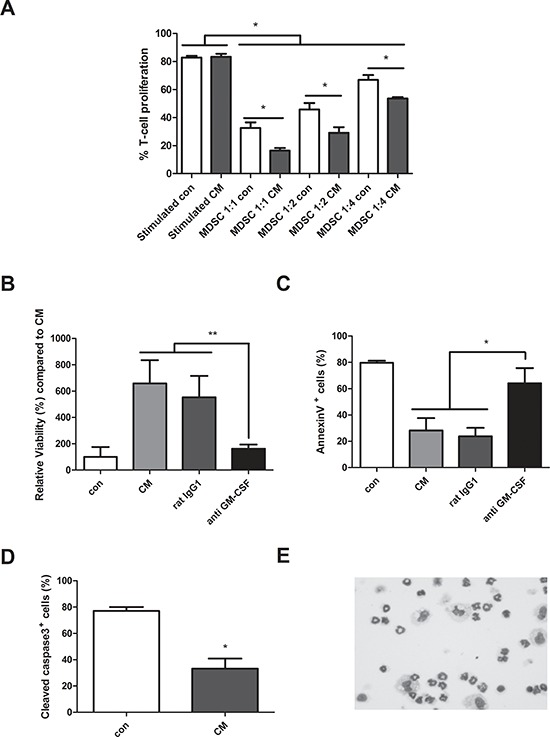
Generation of murine MDSC in myeloma cell conditioned medium **A.** Mouse CD11b^+^ cells were cultured in 5T33MMvt-CM for 3 days at different MDSC/spleen cell ratios. CFSE labeled cells were activated with anti-CD3/CD28 Dynabeads and T cell proliferation was determined by FACS staining (*n* = 3). **B.** CD11b^+^ cells were isolated from the BM of naive C57BL/KaLwRij mice and cultured in 5T33MMvt-CM for 48 h. Viability was measured by CellTiter-Glo assay (*n* = 5). **C and D.** Apoptosis was determined by AnnexinV FITC (*n* = 4) and active Caspase 3 FITC (*n* = 3) with flow cytometry. In some conditions, 10 μg/ml anti GM-CSF or control rat IgG1 was added. **E.** Cytospins of murine CD11b^+^ cells, cultured in 5T33MMvt-CM (2 days) and stained by the May-Grünwald-Giemsa method, are shown. * indicate *p* < 0.05, ** indicate *p* < 0.01 (Mann–Whitney *U*-test). Error bars represent the SD.

As we could observe an induction of MDSC in early stages of MM development, we further wanted to evaluate the underlying mechanisms of MDSC induction *in vitro* stimulated by conditioned medium (CM) derived from 5T33MMvt cells. In first instance, the immunosuppressive capacity was measured as this is the major characteristic of MDSC. Murine CD11b^+^ cells from naïve bone marrow were cultured in presence or absence of 5T33MMvt-CM with total CFSE-labeled spleen cells at different MDSC/spleen cell ratios. T cells were stimulated with anti-CD3/CD28 dynabeads and analyzed for proliferation by flow cytometry. CD11b^+^ cells cultured in the presence of MM-CM were able to significantly decrease T cell proliferation compared to control (Figure 4A). We further investigated the effect of MM-CM on the survival of CD11b^+^ cells. Interestingly, CD11b^+^ cells cultured in CM derived from 5T33MMvt cells, have an increased viability and reduced apoptosis (reduced % Annexin V^+^ and cleaved caspase-3^+^ cells) as compared to CD11b^+^ cells cultured in control medium (Figure 4B–4D). Cytospin stainings showed the presence of both monocytic and polymorphonuclear cells 2 days after incubation with MM-CM for murine CD11b^+^ cells (Figure 4E). This is in accordance with the observation that both sorted Ly6G^low^ and Ly6G^high^ CD11b^+^ cells incubated with 5T33MMvt-CM have a survival benefit (data not shown). Next, we aimed to identify the soluble factor involved in this phenomenon. As GM-CSF, a major survival factor for MDSC was identified in the 5T33MMvt-CM by cytokine array (Supplementary Figure 2), we investigated the effect of a GM-CSF blocking antibody and found that the pro-survival effect of the MM-CM could be abrogated (Figure 4B–4C). Other MDSC survival factors like VEGF and IL-10 are also produced by 5T33MMvt cells, but no effects of anti-VEGF and anti-IL-10 antibodies could be observed (Supplementary Figure 3A–3B).

### Generation of human MDSC in myeloma cell conditioned medium

**Figure 5:**
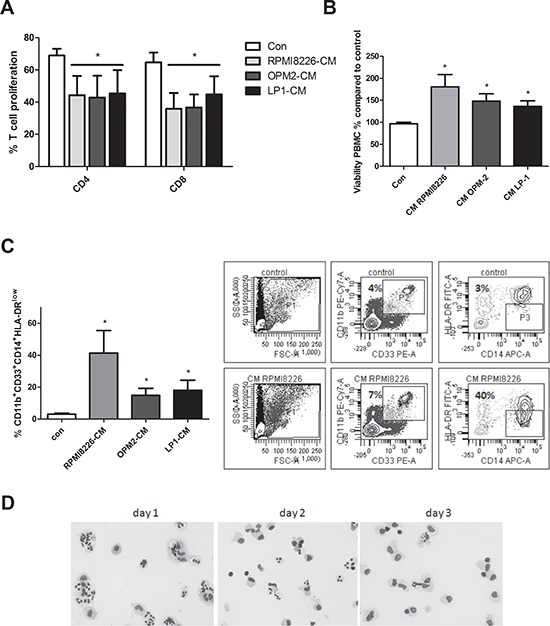
Generation of human MDSC in myeloma cell conditioned medium **A.** Human PBMC were cultured in control medium or HMCL-CM (RPMI8226, OPM2 and LP1) for 6 days and T cell proliferation was determined by FACS staining (*n* = 4). **B.** Peripheral blood mononuclear cells derived from healthy donor blood samples were cultured for 24 h in MM-CM (RPMI8226, OPM2, LP1) and viability was analyzed by CellTiter-Glo assay (*n* = 3). **C.** After 72 h in HMCL-CM, PBMC were analyzed by flow cytometry for MDSC markers CD11b, CD33, CD14 and HLA-DR^low^ (*n* = 4). Gating strategy is shown. **D.** Human CD11b^+^ cells, cultured in RPMI8226-CM (3 days) and stained by the May-Grünwald-Giemsa method, are shown. Bright-field pictures were taken with a Nikon Eclipse 90i microscope at 400x original magnification. * indicate *p* < 0.05 (Mann–Whitney *U*-test). Error bars represent the SD.

We also investigated the induction of human MDSC in MM-CM. Since it is difficult to obtain BM from healthy donors we used peripheral blood mononuclear cells. Total CFSE-labeled PBMC were stimulated with anti-CD3/CD28 dynabeads and cultured in control medium or CM derived from HMCL (RPMI8226, OPM2 and LP1). A significantly decreased CD4^+^ and CD8^+^ T cell proliferation was observed in the cultures with MM-CM (Figure 5A) indicating that an immunosuppressive population is generated in MM-CM. Similar to mouse, an increased viability of total PBMC in the presence of HMCL-CM could be observed (Figure 5B). Furthermore, the presence of human MDSC was analyzed based on CD11b, CD33, HLA-DR, CD14 and CD15 expression. We did not detect differences in the total percentage of CD11b^+^CD33^+^ cells between the control condition and HMCL-CM. However, within the CD11b^+^CD33^+^ population, the percentages of mononuclear MDSC (identified as CD14^+^HLA-DR^low^) were increased compared to control conditions (Figure 5C). Interestingly, human CD11b^+^ cells with a monocytic and polymorphonuclear morphology could be observed 1–2 days after culture in MM-CM. After 3 days of culture, monocytic cells remained while cells with a polymorphonuclear morphology disappeared (Figure 5D). These data indicate that soluble factor(s) secreted by MM cells have the capacity to induce MDSC with T cell suppressive ability. Additionally, we investigated cytokines responsible for this MDSC induction. As GM-CSF is not expressed by human MM cell lines (data not shown), we investigated the effects of anti-VEGF, anti-IL-10 and anti-M-CSF, cytokines which are associated with MDSC expansion and produced by human MM cells. Anti-IL-10 was able to partially decrease the accumulation of CD14^+^HLA-DR^low^ in RPMI8226-CM, however we could not observe this effect in the presence of LP1-CM (Supplementary 3C–3D). Anti-VEGF and anti-M-CSF had no effect on the presence of MDSC.

### Underlying mechanisms for MDSC survival

**Figure 6:**
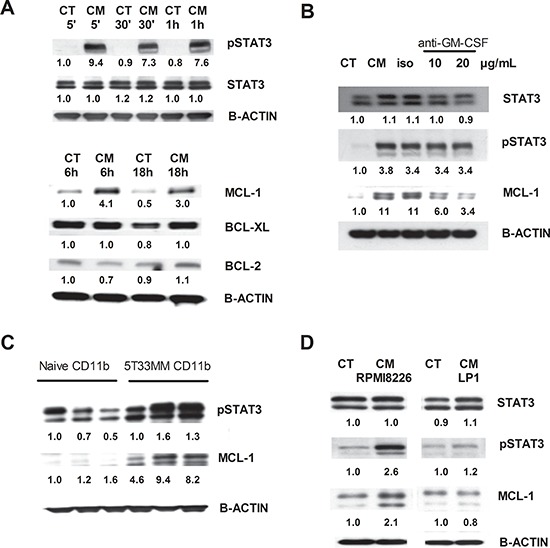
Underlying mechanisms for MDSC survival Western blot analysis for pSTAT3, STAT3, Mcl-1, Bcl-xL and Bcl-2 on mouse CD11b^+^ cells and human CD11b^+^ cells. **A.** Mouse CD11b^+^ cells were incubated for 5 min, 30 min, 1 h, 6 h and 18 h in 5T33MMvt-CM. **B.** Mouse CD11b^+^ cells were incubated with 5T33MMvt-CM in the presence of 10–20 μg/mL anti-GM-CSF for 24 h. **C.** CD11b^+^ cells derived from naive and 5T33MM mice were compared after *in vivo* isolation. **D.** Human CD11b^+^ cells were incubated in RPMI8226-CM and LP1-CM for 18 h. One experiment representing three is shown. The pixel densities of proteins were normalized to B-actin and quantified by ImageJ.

To unravel the mechanism of MM-induced MDSC survival and activity, expression of apoptosis regulating proteins from the Bcl-2 family (Bcl-2, Bcl-xl, Mcl-1) and survival proteins (pSTAT3, STAT3) were investigated by western blot. Mouse CD11b^+^ cells incubated with 5T33MMvt-CM showed a clear increase in pSTAT3 compared to control. Moreover, the anti-apoptotic protein Mcl-1 was clearly increased in 5T33MMvt-CM compared to control-treated cells, while Bcl-2 and Bcl-xl were only slightly higher (Figure 6A). Interestingly, anti-GM-CSF treatment strongly reduced Mcl-1 expression, while leaving pSTAT3 induction unaltered (Figure 6B). In addition, bone marrow CD11b^+^ cells derived from naïve and 5T33MM mice were compared immediately after BM isolation and purification. pSTAT3 as well as Mcl-1 were upregulated in CD11b^+^ cells derived from tumor-bearing mice compared to those from naïve mice (Figure 6C). The results of pSTAT3 and Mcl-1 were confirmed using human cells. CD11b^+^ cells incubated in control or RPMI8226-CM resulted in an increase in pSTAT3 and Mcl-1. For LP1-CM, we only observed a modest increase in pSTAT3 (Figure 6D).

**Figure 7:**
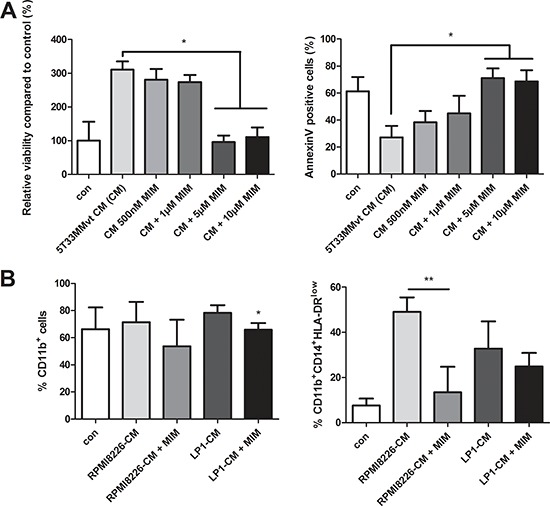
Effect of Mcl-1 targeting by MIM1 on MDSC survival **A.** Murine CD11b^+^ cells were cultured in 5T33MMvt-CM for 48 h with increasing concentrations of the Mcl-1inhibitor MIM1 (MIM). Viability was measured by CellTiter-Glo and apoptosis by AnnexinV staining (*n* = 3). **B.** Human CD11b^+^ cells were incubated with MIM1 in RPMI8226-CM and LP1-CM for 72 h and analyzed for MDSC markers by FACS (*n* = 5). * indicates *p* < 0.05, ** indicate *p* < 0.01 (Mann–Whitney *U*-test). Error bars represent the SD.

To verify the role of Mcl-1 in MDSC survival, murine 5T33MMvt-CM-treated CD11b^+^ cells were incubated with MIM1, a novel Mcl-1 inhibitor [22]. The data clearly showed a decrease in survival, associated with an increase in apoptosis, of the murine CD11b^+^ cells in presence of MIM1 (Figure 7A). Incubation of human CD11b^+^ cells in RPMI8226-CM with MIM1 resulted in a slight decrease in the percentage of CD11b^+^ cells. However, within that population, a significant reduction in the monocytic MDSC subset was observed. MIM1 treatment in presence of LP1-CM resulted in a minor decrease of monocytic MDSC accumulation (Figure 7B). This is in accordance with the absence of Mcl-1 induction by LP1-CM. All these data indicate an important role of Mcl-1 but not pSTAT3 in MDSC survival and accumulation induced by MM soluble factors.

## DISCUSSION

Previous studies showed an important role of MDSC in the regulation of MM cell growth, mainly focusing on their immunosuppressive capacity [18, 19]. In this paper we assessed the presence of MDSC during disease progression, the MDSC-generating and activating potential of MM cells, the underlying mechanism of MDSC survival and the potential of MDSC-targeting *in vivo*. We took advantage of the immunocompetent 5T33MM mouse model to quantify the MDSC population during disease progression. This murine model resembles the human disease closely and allows the ability to investigate the interactions between MM cells and the BM microenvironment [23]. We observed an increase of CD11b^+^ cells in the BM one week after MM cell inoculation, while circulating myeloid cells increased at later stages of the disease. At end-stage, a decline of CD11b^+^ cells was observed in the BM and spleen due to a high expansion of MM cells in these organs. These data are in accordance with observations by Ramachandran et al. who demonstrated a rapid accumulation of CD11b^+^ cells in the BM of double transgenic c-myc/Bcl-xL mice [19]. Furthermore, we observed that the immature myeloid cell subtype increased during disease progression, indicating a block in the differentiation of CD11b^+^ cells. In our previous study, we demonstrated an increased immunosuppressive capacity of MDSC subsets already at early time points during MM progression [20]. All these data indicate that MDSC are important at an early stage of MM development. To investigate the importance of MDSC in MM development, we decided to test an anti-GR1 depleting antibody to target the MDSC population *in vivo*. As 5T33MM cells express GR1, we preferred to use the similar 5TGM1 mouse model in which the tumor cells were negative for GR1 expression (data not shown). In the 5TGM1 model, MDSC expansion in blood, BM and spleen could be observed up to 28 days after MM cell inoculation [24]. Anti-GR1 antibodies bind Ly6G and Ly6C molecules and are extensively described to deplete granulocytic MDSC [25, 26]. However, some controversy exists about the MDSC depleting potential of anti-GR1 antibodies in the BM. Ribechni *et al*. could not observe a depletion of MDSC in the BM and demonstrated an induction of myeloid cell expansion. In addition, they hypothesized that Mcl-1 expression in BM MDSC prevented apoptosis by anti-GR1 antibodies [27]. In contrast to these observations, we observed a significant reduction in tumor load, mainly by depletion of CD11b^+^Ly6G^+^ cells in the BM, and increased CD8^+^ T cell activation. Tumor load in the spleen was also decreased when MDSC were depleted but the effects were less pronounced than in the BM. This is probably due to a more monocytic phenotype of MDSC in the spleen which is unaffected by the anti-GR1 antibody. We previously described that both Ly6G^low^ and Ly6G^high^ cells from the bone marrow of MM-bearing mice have immunosuppressive capacity [20]. While Ly6G^low^ cells are more potent immunosuppressors than Ly6G^high^ cells, Ly6G^high^ cells are the most abundant cell type in the BM (±70%), pointing out that both populations are essential to target in MM disease. Indeed, the chemotherapeutic agent with MDSC-depleting capacity, 5-Fluorouracil, which mainly affects the Ly6G^low^ population also resulted in a significant reduction in tumor load in the 5T33MM model.

It has been shown that currently used anti-MM drugs such as lenalidomide and bortezomib do not alter the MDSC frequency and suppressive function [18]. Also, pomalidomide has minor effects on the immunosuppressive capacity of MDSC [28]. Therefore, the efficacy of immunomodulating therapies could be improved by additional targeting of MDSC. We recently reviewed distinct strategies to target the MDSC population in hematological malignancies [14]. Inhibition could be achieved by deactivation of MDSC (e.g. phosphodiesterase-5 inhibitors, nitro-aspirin, cyclooxygenase-2 inhibitors), inhibition of MDSC differentiation/development (e.g. nitro-bisphophonates) and MDSC depletion (5-Fluorouracil, Gemcitabine, Sunitinib, anti-GR1 antibodies) [29, 30]. Current clinical trials with Sunitinib already show improved clinical outcomes of patients with metastatic renal cell carcinoma [31]. Phosphodiesterase inhibitor Tadalafil entered a phase II clinical trial in MM patients to improve the efficacy of dexamethasone and lenalidomide [32]. The group of Borello *et al.* recently demonstrated a reduction in iNOS and arginase-1 expression in MDSC derived from an end-stage relapsed/refractorty patient treated by Tadalafil, accompanied by a reduction in M-spike [33].

We further wanted to study the underlying mechanisms of MDSC survival induced by MM cells. T cell proliferation assays showed a clear immunosuppressive activity in the presence of MM-CM, demonstrating the generation of an MDSC population *in vitro*. We observed increased viability and reduced apoptosis of murine MDSC cultured in MM-CM. We identified GM-CSF in 5T33MMvt-CM and observed a significant reduction in viability by the use of a GM-CSF blocking antibody. Stromnes and colleagues also demonstrated GM-CSF as an important MDSC survival factor in pancreatic ductal adenocarcinoma [34]. Although human MM cells do not secrete GM-CSF, it is possible that GM-CSF present in the BM microenvironment could influence MDSC generation [35]. Interestingly, we observed an increase in the monocytic (CD11b^+^CD33^+^HLA-DR^low^CD14^+^) MDSC population after administration of MM-CM to PBMC in culture. Based on morphological staining, we found a more monocytic population 3 days after culture of healthy donor PBMC in RPMI8226-CM. This is in accordance with the study of Lechner *et al*. where they characterized cytokine-induced MDSC from normal PBMC *in vitro* [36]. In this study, they described that cytokines GM-CSF, IL-6, VEGF, IL-1β and TNF-α are able to generate human CD33^+^ MDSC. Considering cytokines produced by MM cells, we investigated the involvement of IL-10, M-CSF and VEGF in MDSC survival. In our experiments, IL-10 was partially involved in MDSC accumulation induced by RPMI8226-CM, while VEGF and M-CSF were not involved. In a previous study in MM an induction of CD11b^+^CD14^−^CD33^+^CD15^+^ MDSC was observed when PBMC were cocultured with RPMI8226 cells [18]. We could not observe a generation of CD11b^+^CD33^+^CD15^+^ MDSC in our cultures. However, as recently described as a novel MDSC subtype in MM, we did see some co-expression of CD15 on CD14^+^HLA-DR^low^ cells [37].

The main goal of our research was to understand the underlying mechanism of MDSC survival in the presence of MM cells. Previously, it was demonstrated that S100A9 (MRP 14), a protein abundantly expressed in myeloid cells, is involved in the accumulation of MDSC in MM mouse models [19, 38]. In our study, western blot data clearly showed an increase in STAT3 phosphorylation and Mcl-1 expression after incubation with MM-CM. It has been described that GM-CSF is an important inducer of the JAK/STAT pathway (more specific JAK2, STAT3 and STAT5B) in human neutrophils [39]. Furthermore, STAT3 has been described as an important regulator of MDSC expansion and plays a role in their immunosuppressive capacity through arginase-1 regulation [40]. However, in our study GM-CSF neutralization resulted in a decrease of Mcl-1, while pSTAT3 remained unaffected. In addition, while STAT3 inhibition with AG-490, a JAK-2 tyrosin kinase inhibitor, was not able to abrogate the effect of MM-CM on MDSC proliferation (data not shown), the Mcl-1 inhibitor MIM1 was able to completely abrogate the effect of MM-CM on the viability of murine CD11b^+^ cells. This was accompanied by an increased apoptosis. Furthermore, human CD11b^+^ cells cultured in RPMI8226-CM in the presence of MIM1 showed a decrease in the accumulation of MDSC (CD11b^+^CD33^+^HLA-DR^low^CD14^+^). It has been shown that MIM1 selectively targets the BH3-binding pocket and neutralizes Mcl-1 (not Bcl-xL), thereby inducing capase 3/7 activation and apoptosis in Mcl-1 dependent leukemic cells (IC50 4.2 μM) [41]. Importantly, we demonstrate that Mcl-1 plays a critical role in MDSC survival induced by MM cells. This is in accordance with a recent study in solid tumors [42]. Although in contrast to our study, depletion of granulocytic MDSC did not alter tumor incidence in neuroblastoma, they underline the importance of Mcl-1 in the survival of granulocytic MDSC. It was recently described that Mcl-1 is also important for the survival of regulatory T cells [43]. Taken together, Mcl-1 targeting strategies in MM are not only important for direct anti-tumor effects but also modulate the BM microenvironment including MDSC to enhance the host immune response [44].

In summary, our data indicate an Mcl-1 dependent survival of MDSC in the presence of MM cells. Furthermore, MDSC depletion showed potential as a new treatment option for MM patients. Studies combining anti-MM drugs with MDSC targeting agents, especially in stages of low tumor burden should be further investigated.

## MATERIALS AND METHODS

### Mice

C57BL/KaLwRij mice were purchased from Harlan CPB (Horst, the Netherlands). They were housed and maintained following the conditions approved by the Ethical Committee for Animal Experiments, Vrije Universiteit Brussel (license no. LA1230281). For the 5T33MM mouse model, the 5T33MMvv cell line originated spontaneously in elderly C57BL/KaLwRij mice and have since been propagated *in vivo* by intravenous transfer of the diseased marrow into young syngeneic mice [45].

### Cell lines

The 5T33MMvt and 5TGM1 cell lines resulted spontaneously from cultured 5T33MMvv cells and they grow *in vitro*, independently from BM stroma. 5TGM1 cells were previously modulated to express eGFP [46]. Three well characterized human myeloma cell lines (HMCL), namely RPMI8226, OPM2 and LP1 were selected for our experiments. Murine cells were maintained in RPMI1640 medium (Lonza, Basel, Switzerland) supplemented with penicillin/streptomycin, Na-pyruvate, glutamine, MEM (Gibco, Eggenstein Germany) and 10% fetal bovine serum (Hyclone, UT, USA) at 37% in 5% CO_2_. HMCL were cultured in RPMI1640 medium supplemented with penicillin/streptomycin, glutamine, and 10% fetal bovine serum. Authenticity of HMCLs was regularly confirmed by short-tandem repeat analysis.

### Drugs and blocking antibodies

The following blocking antibodies were used: 10–20 μg/mL anti-mouse GM-CSF blocking antibody (eBioscience, San Diego, CA, USA), 20 μg/mL anti-mouse VEGF, 20 μg/mL anti-mouse IL-10, 10 μg/mL anti-human GM-CSF, 10 μg/mL anti-human VEGF and 20 μg/mL anti-human IL-10 blocking antibodies (R&D systems, Minneapolis, MN, USA). Isotype controls used were 10 μg/mL rat IgG2aK isotype control (eBioscience) and normal goat IgG control (R&D). Mcl-1 inhibitor MIM1 (R&D) was dissolved in DMSO.

### Cell culture and purification

For murine experiments, BM was isolated from naive mice followed by red blood cell lysis. Human healthy donor blood samples were collected after informed consent and all research was approved by the local ethical committee (B.U.N. 143201316382). Peripheral blood mononuclear cells were obtained from whole blood of healthy donors and separated by Ficoll-Hypaque (Nycomed, Lucron Bioproducts, De Pinte, Belgium) gradient centrifugation. CD11b^+^ cells were positively selected by the use of CD11b MACS beads (Miltenyi Biotec, Bergisch Gladbach, Germany) according to the manufacturer's instructions.

### Flow cytometry

The antibodies used for murine experiments were CD11b-FITC, Ly6G-PECy7, Ly6C-APC, CD3-PECy7, CD4-APC, CD8-FITC, IFNγ-PECy7 and isotype controls. Tumor load in the 5T33MM model was determined by the use of anti-idiotype (3H2) monoclonal antibody (IgG1) and APC-labeled rat anti-mouse IgG1 antibody (secondary step). The development of 3H2 was described previously [23]. The following antibodies were used for human experiments: CD4/CD8-Alexa Fluor^®^647, CD15-Pacific Blue™, CD33-PE, CD11b-PECy7, HLA-DR-FITC, CD14-APC and isotype controls. All antibodies (except 3H2 and IFNγ-PECy7) were obtained from Biolegend (Biolegend, San Diego, CA, USA). IFNγ-PECy7 was derived from Becton Dickinson. Flow cytometric data were acquired using FACS Canto or Fortessa (Becton Dickinson, Franklin Lakes, NJ, USA) and analyzed by FACS Diva Software (BD).

### Viability and apoptosis assay

Cell viability was measured by the use of a CellTiter-Glo^®^ assay (Promega, Madison, WI, USA). Apoptosis was quantified by AnnexinV-FITC staining (Becton Dickinson) and active Caspase-3-FITC staining (Becton Dickinson), followed by flow cytometric analysis.

### Cytospin staining

Cytospins were made and stained by the May-Grünwald-Giemsa method. Bright-field pictures were taken with a Nikon Eclipse 90i microscope at 400x original magnification (Nikon France SAS, Champigny-Sur-Marne, France).

### Preparation conditioned medium

Conditioned medium (CM) was prepared from 5T33MMvt cells or HMCL (RPMI8226, OPM2, LP1) cultured for 48 h in RPMI1640 medium (10% FCS) at a concentration of 10^6^/mL. For experiments, conditioned medium was diluted ½ in fresh RPMI1640 medium (10% FCS). Ten times concentrated conditioned medium with Centriprep (Millipore, Billerica, MA, USA) was used to detect cytokines with the mouse cytokine array panel A (R&D sytems, Minneapolis, MN, USA) according to manufacturer's instructions.

### T cell suppression assay

For murine experiments, cells were isolated from spleen of healthy mice followed by red blood cell lysis. Cells were stained by CFSE (0.1 μM) (Invitrogen, Carlsbad, CA) for 10–15 min at 37°C, centrifuged and resuspended in RPMI1640 medium supplemented with 10% HEPES (Sigma, St Louis, MO, USA) and 20 μM β-mercaptoethanol (Sigma). After 20–30 min incubation at room temperature, they were cultured in a 96-well plate (2 × 10^5^ cells/well). T cells were stimulated with 2 μL of CD3/CD28 Dynabeads (Invitrogen) and cultured for 3 days in the presence or absence of CD11b^+^ cells at different ratios. Cells were cultured in RPMI1640 medium (10% FCS) or conditioned medium of 5T33MMvt cells. Proliferation was analyzed by flow cytometric CFSE dye dilution after CD3 staining. For human samples, total peripheral blood mononuclear cells were labeled with CFSE (0.3 μM), stimulated with 2 μL CD3/CD28 Dynabeads and analyzed 6 days after incubation in HCML-CM at 37°C. Cells were labeled for CD4 and CD8, and analyzed by flow cytometry.

### Western blot

CD11b^+^ cells were cultured in 5T33MMvt-CM or HMCL-CM, at a density of 10^6^/mL and collected at indicated time points. Western blot was performed as described previously [47] using following antibodies: STAT3, pSTAT3, Mcl-1, Bcl-2, Bcl-xL and B-actin (Cell Signaling Technology, Boston, MA). The pixel densities of proteins were quantified by ImageJ.

### *In vivo* treatment with anti-GR1 and 5-Fluorouracil

In the first experiment, naïve mice were intraperitoneally injected with one single injection of the GR1 neutralizing antibody (200 μg/mouse, RB6–8C5) obtained from BioXCell (West Lebanon, NH). Two days after antibody administration, the percentage of CD11b^+^ and Ly6G^+^ cells in the BM was analyzed by flow cytometry. As 5T33MM cells express GR1, we preferred the similar 5TGM1 mouse model in which the tumor cells were negative for GR1 expression (data not shown). 5TGM1 mice were intravenously inoculated at 1 × 10^6^ cells/mouse. One day later, treatment was started with 200 μg GR-1 neutralizing antibody every two days. When first mice showed sign of disease, all mice were sacraficed and analyzed for tumor load at day 39. The percentage of 5TGM1-GFP^+^ cells and IFNγ expressing CD8^+^ cells were determined by flow cytometry in the BM and spleen. Additionally, M-spike was measured by means of serum electrophoresis.

To investigate the effect of 5-Fluorouracil (5FU), 5T33MM mice were intraperitoneally injected with one single injection of 5FU (Sigma), 4 days after 5T33MMvv cell inoculation. Four days after 5FU administration, the percentage of CD11b, Ly6G and Ly6C in the BM and spleen was analyzed by flow cytometry. In a second experiment, 5T33MM mice were treated with 5FU on day 4 and analyzed for tumor load at day 17. The percentage of idiotype positive cells (3H2^+^) was determined by flow cytometry in the BM and spleen. Additionally, M-spike was measured by means of serum electrophoresis.

### Statistics

Statistical analysis was done using GraphPad Prism 5 software. All data represent the mean ± standard deviation (SD), and results were analyzed using the Mann-Whitney *U* test. *p* < 0.05 (*), *p* < 0.01 (**) and *p* < 0.001 (***) were considered statistically significant.

## SUPPLEMENTARY FIGURES


