# Supplementary material for: Multiple myeloma induces Mcl-1 expression and survival of myeloid-derived suppressor cells
Source: Oncotarget. 2015 Mar 23;6(12):10532–47. doi: 10.18632/oncotarget.3300 (PMC4496373; doi:10.18632/oncotarget.3300)
Supplement: Supplementary file 1 [file oncotarget-06-10532-s001.pdf]

SUPPLEMENTARY FIGURES

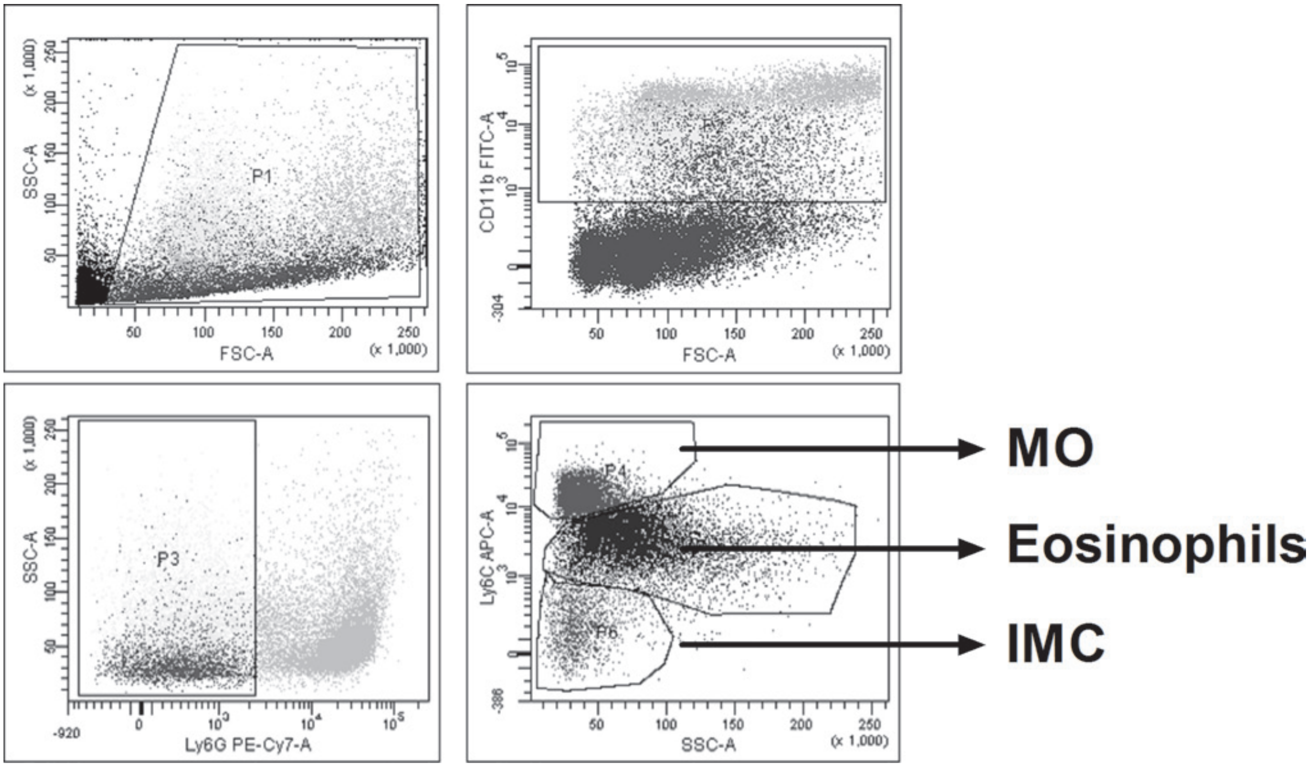

Supplementary Figure S1: MDSC gating strategy. Gating strategy for MDSC during tumor progression based on CD11b, Ly6G and Ly6C by flow cytometry.

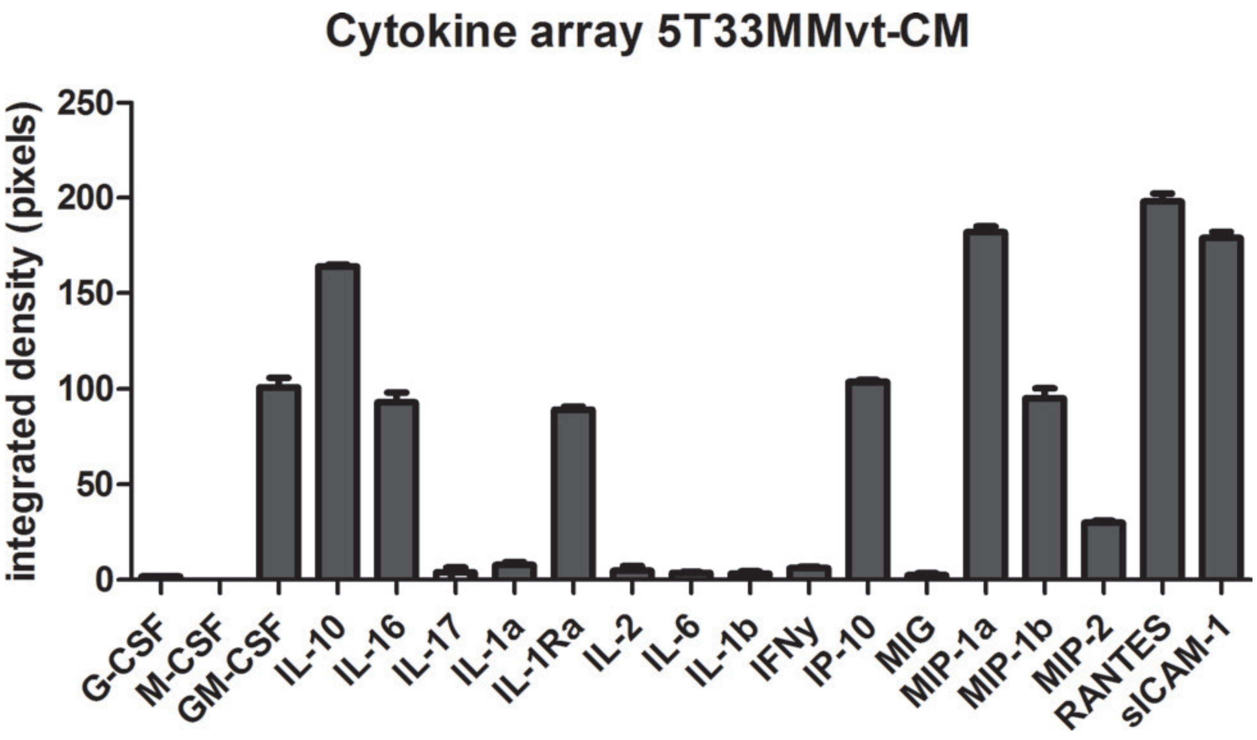

Supplementary Figure S2: Cytokine array for 5T33MMvt-CM. The pixel densities of proteins in the cytokine array (panel A, R&D systems) were quantified by ImageJ.

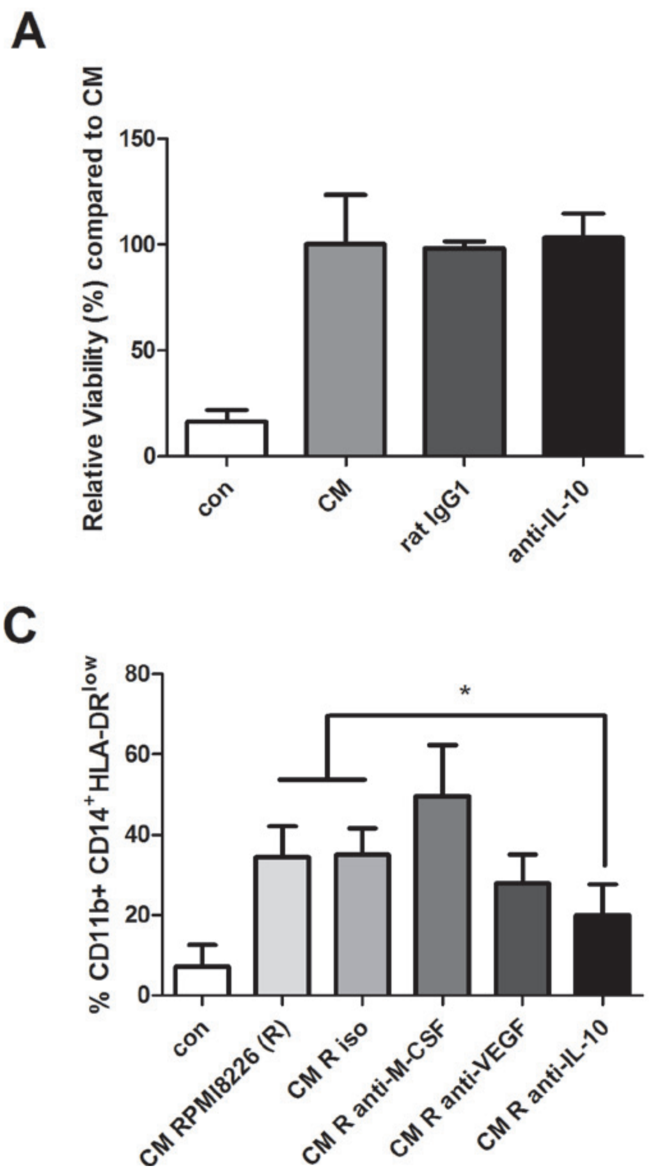

Supplementary Figure S3: Effect of anti-IL-10, anti-VEGF and anti-M-CSF on murine CD11b^{+} cells and human PBMC. A and B. CD11b^{+} cells were isolated from the BM of naive C57BL/KaLwRij mice and cultured in 5T33MMvt-CM for 48 h. Cells were cultured in the presence of 20 µg/mL anti-IL-10 or anti-VEGF antibodies and viability was measured by CellTiter-Glo assay ($n = 3$). **C and D.** Peripheral blood mononuclear cells derived from healthy donor blood samples were cultured in RPMI8226-CM and LP1-CM for 72 h. Cells were incubated with 10 µg/mL VEGF blocking antibody (aVEGF), 20 µg/mL IL-10 blocking antibody (aIL-10) or isotype control. Cells were analyzed by flow cytometry for human MDSC markers CD11b, CD33, CD14, HLA-DR^{low} and CD15 ($n = 4$). *indicate $p < 0.05$ (Mann-Whitney U-test). Error bars represent the SD.
